# Seven‐Day Patch ECG Monitoring During National Insurance Health Checkup Efficiently Detected Silent Atrial Fibrillation in Individuals Aged 75 Years and Older

**DOI:** 10.1111/anec.70092

**Published:** 2025-06-03

**Authors:** Miho Miyoshi, Nozomi Kodama, Hiroki Sato, Kazuhiro Masutomo, Hitoshi Kamiunten, Tetsuji Shinohara, Naohiko Takahashi

**Affiliations:** ^1^ Department of Cardiology and Clinical Examination Faculty of Medicine, Oita University Oita Japan; ^2^ Department of Cardiology Usuki Cosmos Hospital Oita Japan; ^3^ Department of Cardiology Kitsuki City Yamaga Hospital Oita Japan

**Keywords:** health checkup, seven‐day patch ECG monitoring, silent atrial fibrillation

## Abstract

**Background:**

It is unclear to what extent silent atrial fibrillation (AF) is present in subjects previously undiagnosed with AF. The recently popular 7‐day patch electrocardiography (ECG) monitoring may help answer this question.

**Methods:**

In the Kitsuki and Usuki cities in Oita Prefecture, Japan, a study was conducted among subjects who underwent 7‐day patch ECG monitoring (Heartnote) for silent AF screening during the national insurance health checkup between June and November 2023. Subjects were (1) 65–74 years old and have ≥ 1 of the following risk factors: hypertension, diabetes mellitus, stroke, transient ischemic attack, and underlying heart disease (heart failure and/or previous myocardial infarction) and (2) 75 years and older.

**Results:**

A total of 571 subjects (307 females and 264 males, mean age 75.3 ± 5.4 years) were analyzed. Silent AF was detected in 16 out of 571 subjects (2.8%). Among those aged 75 years or older, silent AF was detected in 15 out of 291 subjects (5.2%). In multivariate analysis, among age, body mass index (BMI), hypertension, diabetes, stroke, and underlying heart disease, only age was the independent predictor of silent AF detection (odds ratio: 1.16, 95% confidence interval: 1.06–1.28, *p* < 0.01).

**Conclusions:**

Seven‐day patch ECG monitoring during the national insurance health checkup efficiently detected silent AF in individuals aged 75 years and older.

## Graphical abstract

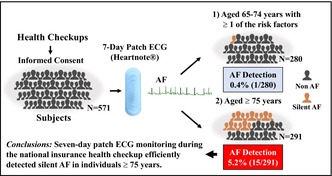

## 1 Introduction

Atrial fibrillation (AF) is one of the most common arrhythmias observed in clinical practice. Regarding the prevalence of AF in the general population in Japan, Inoue et al. reported an overall prevalence of AF of 0.56%, using data from periodic health examinations in 2003 (Inoue et al. 2009). Among 630 subjects, 138 were aged 40 years or older, and the prevalence rate of AF increased with age, reaching 4.4% for men and 2.2% for women aged 80 years or older (Inoue et al. 2009). The authors suggested that the overall prevalence of AF in Japan is approximately two‐thirds of that in the United States (Inoue et al. 2009). In Tama City, Japan, 12‐lead electrocardiography (ECG) has been included as an essential examination during health checkups for diagnosing AF since 2008 (Kodani et al. 2019). The prevalence of AF was 0.8% (1.7% in men and 0.2% in women) in 2008, and 1.4% (2.9% in men and 0.4% in women) in 2015 (Kodani et al. 2019). When age was divided into 10‐year classes, the prevalence of AF in age groups 50–59, 60–69, and 70–79 years was 0.3%, 2.4%, and 4.1% in men and 0%, 0.3%, and 0.7% in women, respectively, in 2015 (Kodani et al. 2019). Thus, the prevalence of AF was higher in the elderly people (Inoue et al. 2009; Kodani et al. 2019). It should be noted that in these reports (Inoue et al. 2009; Kodani et al. 2019), 12‐lead ECG was used to assess the prevalence of AF, indicating that most AF cases are persistent or permanent. There are probably as many patients with paroxysmal AF as those with persistent or permanent AF (Akao et al. 2013). When AF is paroxysmal and asymptomatic, diagnosis using conventional 12‐lead ECG is difficult and requires prolonged ECG monitoring. Recently, long‐term ECG monitoring has become popular for the detection of AF (Barrett et al. 2014; Gladstone et al. 2021; Okubo et al. 2022; Steinhubl et al. 2018; Turakhia et al. 2015). It is important to determine the number of patients with silent AF among those who have been undiagnosed with AF. To address this question, we conducted a 7‐day patch ECG monitoring during the national insurance health checkup of subjects residing in Kitsuki and Usuki cities, Oita Prefecture.

## 2 Methods

The data supporting our findings of this study are available from the corresponding author upon reasonable request.

### 2.1 Subject Selection

In the Kitsuki and Usuki cities in Oita Prefecture, Japan, a study was conducted among subjects who underwent 7‐day patch ECG monitoring (Heartnote) for silent AF screening during the national insurance health checkup between June and November 2023. Subjects were (1) 65–74 years old and have ≥ 1 of the following risk factors: hypertension, diabetes mellitus, stroke, transient ischemic attack, underlying heart disease (heart failure and/or previous myocardial infarction) and (2) 75 years and older. Subjects previously diagnosed with AF were excluded. All subjects provided written informed consent.

### 2.2 Seven‐Day Patch ECG Monitoring

**FIGURE 1:**
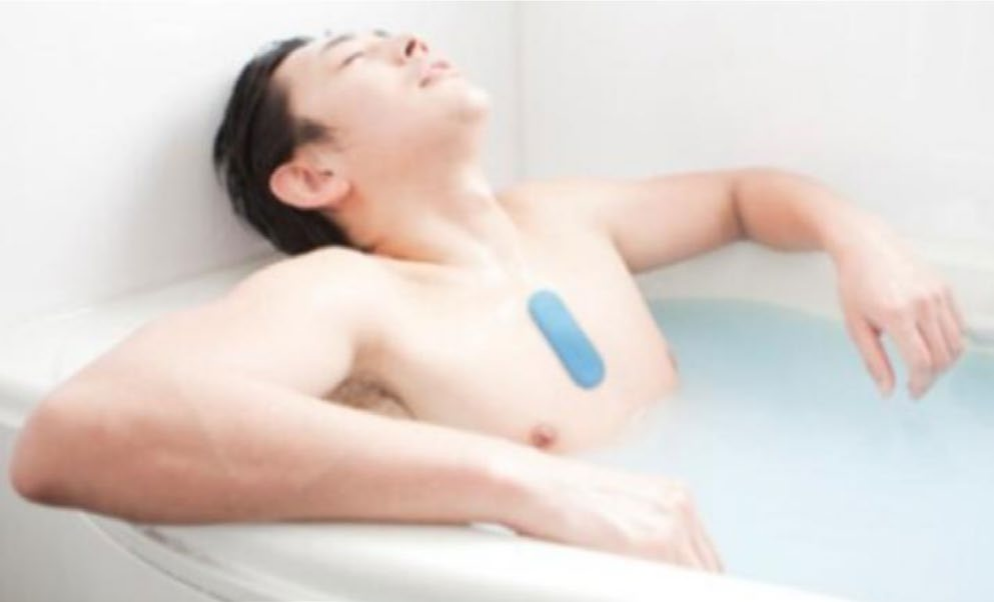
Image of Heartnote. The Heartnote is placed over the precordial region. Images courtesy of JSR Corporation, Tokyo, Japan.

We used a patch ECG (Heartnote, JSR Corporation, Figure 1). The sensor was flexible, codeless, electrode‐integrated, waterproof, measuring 100 mm in length, 30 mm in width, and 5 mm in thickness, and weighing 12 g. The sensor was attached to the upper chest wall with adhesive tape, and the ECG was continuously recorded at 256 Hz and triaxial acceleration at 32 Hz for 7 days. The sensors were attached to subjects, collected after measurements, and returned to JSR Corporation, where stored data were extracted, all QRS complexes in the ECG were detected, noise and arrhythmia types were annotated, and annotated R–R interval time series data were generated (Hayano and Yuda 2022a, 2022b). These processes were performed on a long‐term Holter ECG analysis viewer (NEY‐HEA3000, Nexis Co. Ltd.) using a Holter analyzer program (JMDN 36827012, Nexis Co. Ltd.), approved by the Japanese Ministry of Health, Labour, and Welfare (Medical device approval number 228AGBZX00099000). To annotate the cardiac rhythms, the analyzer program classified the QRS complexes using the standard cycle length criteria for supraventricular ectopic heartbeats, grouped them by QRS morphology, and labeled the groups according to the type of arrhythmia. The results of the automated analysis were reviewed and edited by skilled technicians, and a morphological classification table was provided to the medical doctors for confirmation. The long‐term R–R interval time series thus generated was provided for this study. AF was defined as an irregular rhythm without P‐waves recorded for at least 30 s. All AF diagnoses were confirmed and reviewed by experienced cardiologists (M.M., N.K., and N.T.).

### 2.3 Statistical Analysis

Continuous variables were summarized as mean ± standard deviation, while categorical variables were presented as frequencies and percentages. The characteristics of subjects diagnosed with AF and those without AF were compared using the unpaired t‐test or chi‐squared test, as appropriate. To investigate the relationship between age and the detection rate of AF, we conducted an analysis by categorizing subjects into age groups. The study population was divided into four age groups: 65–69, 70–74, 75–79, and 80 years and older. Subjects' characteristics by age groups were compared using one‐way analysis of variance or Wilcoxon rank‐sum test, as appropriate. The relationship between age groups and the detection rate of AF was examined using the univariate logistic regression analysis. Multivariate logistic regression analysis was used to assess independent predictors of newly diagnosed AF. In addition to the definition of AF as an irregular rhythm without P‐waves lasting at least 30 s, we also evaluated the detection rates of AF using alternative duration thresholds of more than 3 min and more than 6 min. Statistical significance was set at *p* < 0.05. All statistical analyses were performed using the JMP statistical software (JMP version 14.2, SAS, Cary, NC, USA).

## 3 Results

### 3.1 Study Population

**FIGURE 2:**
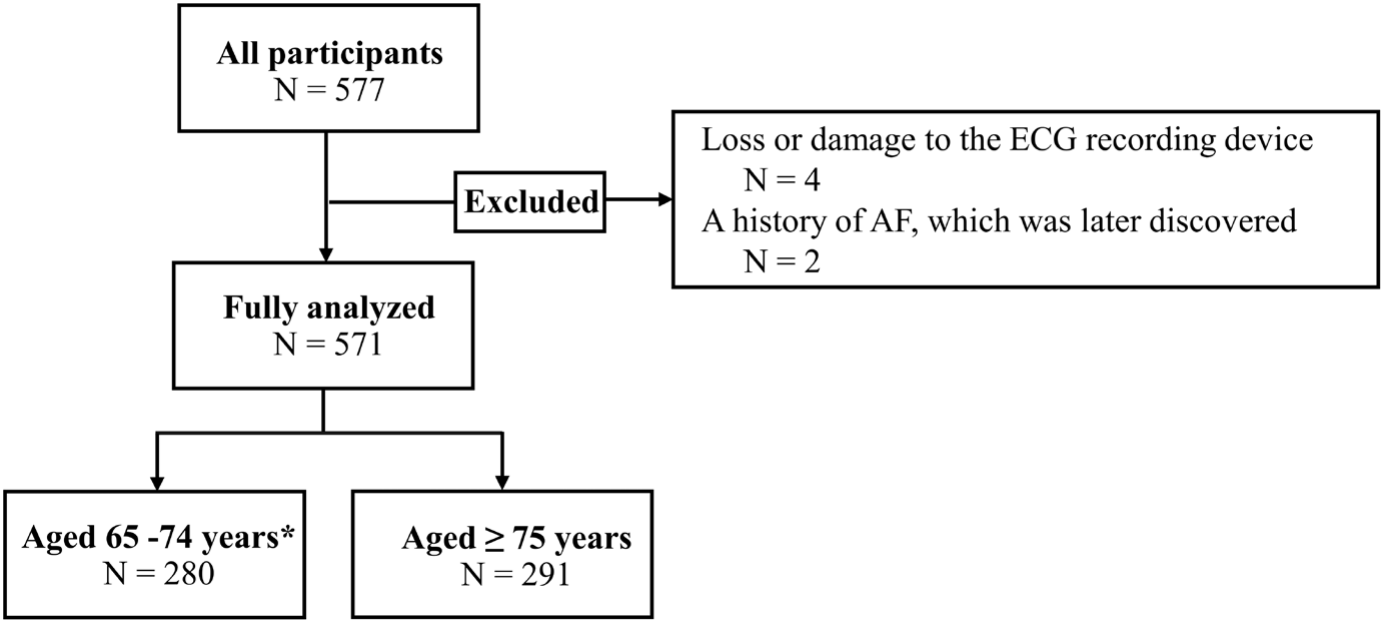
Flow diagram. Subjects who have never been diagnosed with AF during a previous health checkup are included in the study. The inclusion criteria are (1) those aged 65–74 years who are at a high risk of stroke if they have AF* and (2) all subjects aged 75 years or older. *Hypertension, diabetes mellitus, stroke, transient ischemic attack, and underlying heart disease (heart failure, and previous myocardial infarction).

A flow diagram of the study subjects is shown in Figure 2. Seven‐day patch ECG monitoring was conducted on 577 subjects who provided informed consent. Six subjects whose ECG recordings were not available for analysis were excluded. Reasons for exclusion were loss or damage to the ECG recording device in four cases and a history of AF, which was later discovered in two cases. A total of 571 subjects were analyzed.

### 3.2 Basic Characteristics

The baseline characteristics of the 571 subjects (307 females, 264 males, aged 75.3 ± 5.4 years) are described in Table 1. The mean body mass index (BMI) was 23.6 ± 3.2 kg/m^2^. The most common comorbidity was hypertension (65.4%), followed by dyslipidemia (43.0%) and diabetes mellitus (15.1%). Underlying heart disease was observed in 6.6%, while prior stroke was observed in 3.2%. The mean systolic/diastolic blood pressure was 130.2 ± 16.8/75.0 ± 9.4 mmHg, and the mean heart rate was 67.0 ± 10.0 beats/min. The comparison of the basic characteristics between the AF and non‐AF groups showed no significant difference except for age (79.5 ± 4.5 vs. 75.2 ± 5.4 years old, *p* = 0.002).

**TABLE 1:** Baseline characteristics and comparisons between AF and non‐AF groups.

	Total (*n* = 571)	AF (*n* = 16)	Non‐AF (*n* = 555)	*p*
Age	75.3 ± 5.4	79.5 ± 4.5	75.2 ± 5.4	0.002
Male, *n* (%)	264 (46.1)	10 (62.5)	254 (45.8)	0.186
BMI (kg/m^2^)	23.6 ± 3.2	24.6 ± 3.4	23.6 ± 3.2	0.222
Hypertension, *n* (%)	373 (65.4)	11 (68.8)	362 (65.3)	0.785
Diabetes, *n* (%)	86 (15.1)	2 (12.5)	84 (15.2)	0.771
Dyslipidemia, *n* (%)	245 (43.0)	8 (50.0)	237 (42.8)	0.561
Stroke, *n* (%)	18 (3.2)	1 (6.3)	17 (3.1)	0.472
Underlying heart disease, *n* (%)	38 (6.6)	2 (12.5)	36 (6.5)	0.341
Systolic blood pressure (mmHg)	130.2 ± 16.8	132.4 ± 15.5	130.1 ± 16.8	0.597
Diastolic blood pressure (mmHg)	75.0 ± 9.4	72.5 ± 9.2	75.1 ± 9.4	0.273
Heart rate (beats/min)	67.0 ± 10.0	65.3 ± 9.0	67.0 ± 10.1	0.514
HbA1c (%)	6.03 ± 0.48	6.11 ± 0.57	6.02 ± 0.48	0.507

*Note:* Continuous variables are presented as mean ± standard deviation and compared using an unpaired t‐test. Categorical variables are presented as frequency (percentage) and compared using the chi‐squared test.

Abbreviations: AF, atrial fibrillation; BMI, body mass index.

The baseline characteristics by age groups are presented in Table 2. The prevalence of comorbidities did not differ significantly among the age groups. The proportion of underlying heart disease was lower in the older group; however, the difference was not statistically significant (*p* = 0.058). Diastolic blood pressure was significantly lower in the older age groups (*p* < 0.001).

**TABLE 2:** Baseline characteristics and comparisons across age groups.

	65–69 y. o. (*n* = 102)	70–74 y. o. (*n* = 179)	75–79 y. o. (*n* = 182)	80 y. o. – (*n* = 108)	*p*
Age	67.7 ± 0.2	72.7 ± 0.1	77.3 ± 0.1	83.4 ± 0.2	< 0.001
Male, *n* (%)	40 (39.2)	94 (52.5)	79 (43.4)	51 (47.2)	0.139
BMI (kg/m^2^)	23.8 ± 3.4	23.7 ± 3.2	23.7 ± 3.3	23.3 ± 2.8	0.764
Hypertension, *n* (%)	66 (65.4)	112 (62.6)	118 (64.8)	77 (72.0)	0.441
Diabetes, *n* (%)	18 (17.7)	33 (18.4)	23 (12.6)	12 (11.1)	0.233
Dyslipidemia, *n* (%)	46 (45.1)	73 (40.8)	74 (40.7)	52 (48.2)	0.549
Stroke, *n* (%)	3 (2.9)	8 (4.5)	6 (3.3)	1 (0.93)	0.424
Underlying heart disease, *n* (%)	3 (2.9)	11 (6.2)	11 (6.0)	13 (12.0)	0.058
Systolic blood pressure (mmHg)	131.2 ± 19.2	129.5 ± 15.7	129.4 ± 16.4	131.6 ± 16.9	0.617
Diastolic blood pressure (mmHg)	78.5 ± 10.6	75.7 ± 9.5	73.9 ± 8.6	72.5 ± 8.3	< 0.001
Heart rate (beats/min)	68.8 ± 1.0	66.5 ± 0.7	67.2 ± 0.7	65.4 ± 1.0	0.095
HbA1c (%)	6.1 ± 0.5	6.0 ± 0.5	6.0 ± 0.5	6.0 ± 0.4	0.728

*Note:* Continuous variables are presented as mean ± standard deviation and compared using one‐way analysis of variance. Categorical variables are presented as frequency (percentage) and compared using Wilcoxon rank‐sum test.

Abbreviations: BMI, body mass index; y. o., years old.

### 3.3 AF Detection Rate by Age Group

**FIGURE 3:**
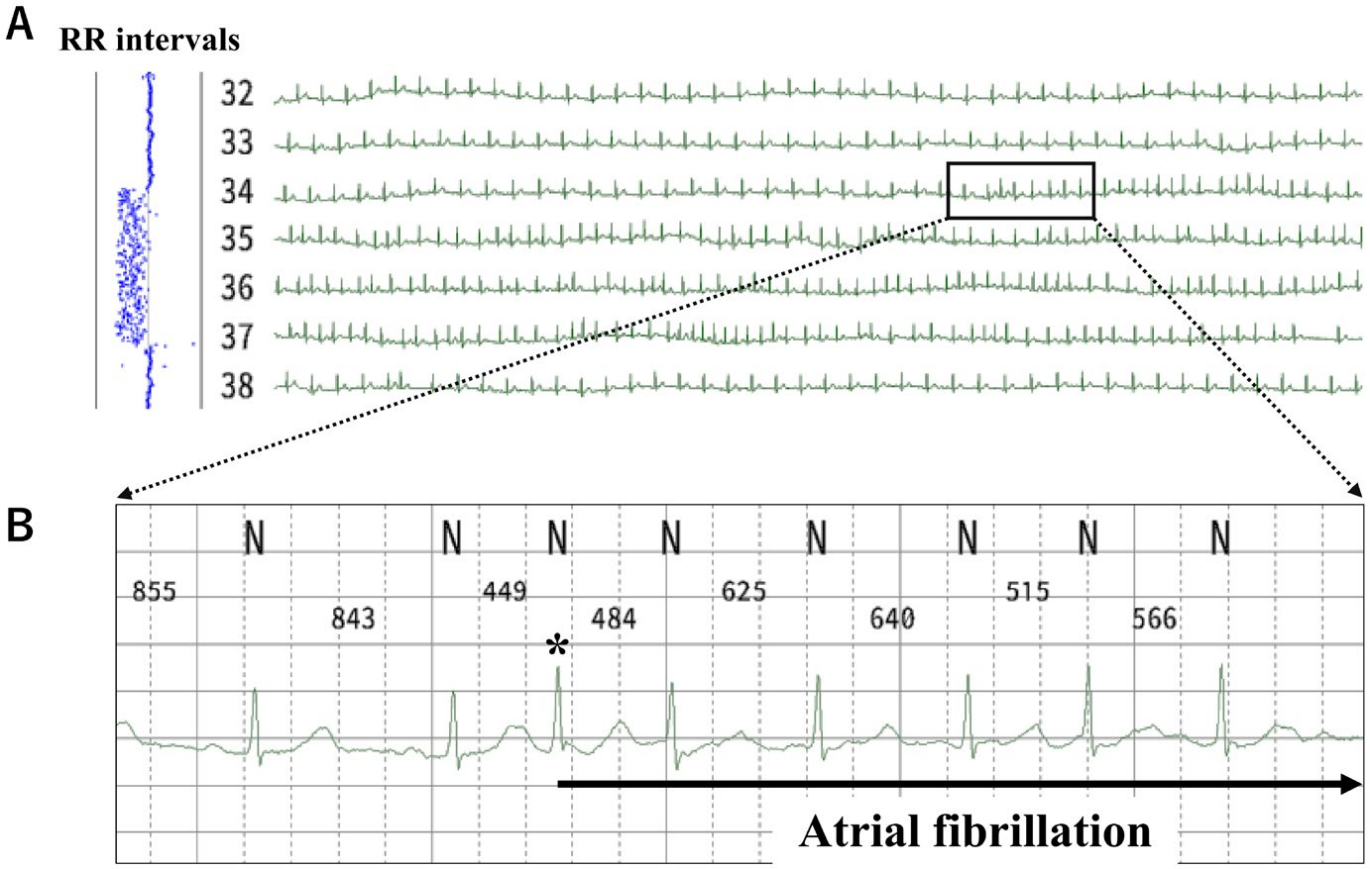
Detection of AF by Heartnote. Representative case (Case 1). (A) Compressed ECG. AF is initiated within the frame. The RR intervals are completely irregular at the same time (left side). (B) An expanded ECG in frame A. R–R intervals are automatically measured; AF started and continued for 192 s. *indicates the beat that initiated AF.

Table 3 shows the number and detection rate of silent AF. Figure 3 shows the detection of silent AF in a 75‐year‐old woman. The frame in Figure 3A shows the initiation of AF. In the expanded ECG, shown in Figure 3B, premature atrial contraction (indicated by *) triggers AF lasting for 192 s. During AF, the RR interval was irregular (Figure 3A, left).

**TABLE 3:** Number and rate of detection of silent AF by age groups.

	65–69 y. o. (*n* = 102)	70–74 y. o. (*n* = 179)	75–79 y. o. (*n* = 182)	80 y. o. – (*n* = 108)	Total (*n* = 571)	*p*
AF (%)	0 (0.0)	1 (0.6)	10 (5.5)	5 (4.6)	16 (2.8)	0.006
Non‐AF (%)	102 (100.0)	178 (99.4)	172 (94.5)	103 (95.4)	555 (97.2)	

Abbreviations: AF, atrial fibrillation; y. o., years old.

**FIGURE 4:**
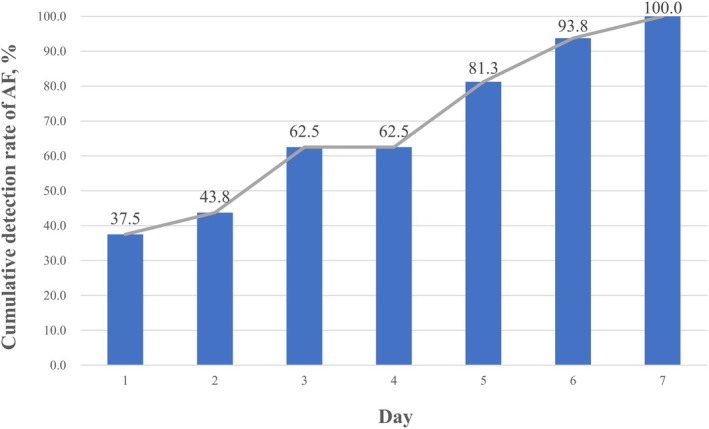
Cumulative detection rate of AF. AF was first detected in 6 patients on Day 1 (cumulative rate: 37.5%), 2 on Day 2 (cumulative rate: 43.8%), 3 on Day 3 (cumulative rate: 62.5%), 0 on Day 4, 3 on Day 5 (cumulative rate: 81.3%), 2 on Day 6 (cumulative rate: 93.8%), and 1 on Day 7 (cumulative rate: 100%).

In total, silent AF was detected in 16 subjects (2.8%). When the subjects were divided by age (65–69, 70–74, 75–79, and 80 years and older), silent AF was detected in only one of 179 subjects (0.6%) in the 70–74 years group, while it was detected in 10 of 182 (5.5%) in the 75–79 years group and 5 of 108 in the 80 years and older group. No AF cases were detected in subjects aged 65–69 years. The detection rates of AF significantly differed across the age groups (*p* = 0.006). In our additional analysis, of the 16 patients diagnosed with AF (≥ 30 s) by the current definition, AF (≥ 3 min) was observed in 13 patients and AF (≥ 6 min) in 11 patients. Further investigation is needed into the duration of AF requiring treatment. Figure 4 illustrates the cumulative detection rate of AF. AF was first detected in 6 patients on Day 1 (cumulative rate: 37.5%), 1 on Day 2 (cumulative rate: 43.8%), 3 on Day 3 (cumulative rate: 62.5%), none on Day 4, 3 on Day 5 (cumulative rate: 81.3%), 2 on Day 6 (cumulative rate: 93.8%), and 1 on Day 7 (cumulative rate: 100%). The results were similar when the study subjects were limited to those aged 75 years or older (15 subjects with AF).

### 3.4 Multivariate Analysis

The results of multivariate analysis to predict silent AF detection are shown in Table 4. Among age, BMI, hypertension, diabetes, stroke, and underlying heart disease, only age was the independent factor in predicting silent AF detection (odds ratio: 1.16, 95% confidence interval: 1.06–1.28, *p* = 0.002).

**TABLE 4:** Multivariate logistic analysis to predict silent AF.

	OR [95% CI]	*p*
Age	1.16 [1.06–1.28]	0.002
BMI	1.12 [0.95–1.31]	0.167
Hypertension	1.03 [0.30–3.53]	0.955
Diabetes	0.93 [0.19–4.42]	0.922
Stroke	3.25 [0.37–28.7]	0.288
Underlying heart disease	1.60 [0.36–8.11]	0.569

Abbreviations: AF, atrial fibrillation; BMI, body mass index; CI, confidence interval; OR, odds ratio.

### 3.5 Details of Silent AF Subjects

A list of the 16 patients with silent AF is presented in Table 5. AF burden ranged from 0.66 min to 3708 min (mean 512 ± 1065 min). All 16 subjects had a CHADS_2_ score of 1 or higher, with a mean CHADS_2_ score of 1.8 ± 0.7. The mean CHA_2_DS_2−_VASc score was 2.2 ± 0.8. According to predetermined rules, these 16 patients were referred to Oita University Hospital (N.T. or T.S.), Kitsuki City Yamaga Hospital (H.K.), or Usuki City Cosmos Hospital (K.M.). Except for one subject who refused referral (Case 10), 15 subjects visited the hospitals. N.T., T.S., H.K., and K.M. decided on a treatment plan for each patient according to the guidelines of the Japanese Circulation Society (Iwasaki et al. 2025; Ono et al. 2022). Oral anticoagulation was initiated in all 15 subjects, and catheter ablation was performed in three subjects (Cases 4, 9, and 11). For Case 10, repeated efforts were made to advise the patient to seek medical attention and initiate treatment, but the patient did not present for the consultation, and treatment could not be initiated.

**TABLE 5:** List of silent AF subjects.

Case no	Age	Sex	CHADS_2_ score	CHA_2_DS_2_‐VASc score	AF Burden, min (%)	Treatment after diagnosis of AF	
						OAC	Ablation
1	75	F	2	3	5.9 (0.06)	+	−
2	76	F	1	2	0.84 (0.01)	+	−
3	78	F	3	4	553.7 (5.6)	+	−
4	78	F	2	3	127.3 (1.3)	+	+
5	80	F	2	3	12.1 (0.3)	+	−
6	83	F	2	3	0.66 (0.01)	+	−
7	71	M	1	2	2434.8 (24.2)	+	−
8	76	M	2	2	48.4 (3.5)	+	−
9	77	M	1	1	8.9 (0.1)	+	+
10	78	M	2	2	160.4 (2.0)	−	−
11	78	M	4	4	6.7 (0.08)	+	+
12	78	M	2	2	3708.1 (36.4)	+	−
13	79	M	2	2	4.7 (0.05)	+	−
14	81	M	1	1	1079.2 (10.0)	+	−
15	88	M	2	2	16.7 (0.17)	+	−
16	89	M	1	1	23.6 (0.2)	+	−

Abbreviations: AF, atrial fibrillation; F, female; M, male; OAC, oral anticoagulant.

## 4 Discussion

In our study, we targeted subjects in national insurance health checkups who had never been previously diagnosed with AF and detected silent AF in 16 subjects (2.8%). Notably, AF was detected in 5.2% of subjects aged 75 years or older. Conversely, silent AF was detected in only 0.6% of at‐risk subjects aged 70‐74 with risk factors, and no cases of silent AF were detected in subjects aged 65–69 years. In the multivariate analysis, among age, BMI, hypertension, diabetes, history of stroke, and underlying heart disease, only age emerged as an independent predictor of silent AF. The results of our study suggest that being 75 years old or older was a significant factor in predicting silent AF. The question naturally arises as to whether 7 days is sufficient for AF detection. As shown in Figure 4, AF was most frequently detected on Day 1, in 6/16 patients. On the other hand, six patients were detected after Day 5. This observation suggests that 7‐day patch ECG monitoring is extremely useful in detecting AF compared to the conventional 24‐h Holter ECG and suggests that some number of patients may have AF detected after the 8th day. In fact, Liu et al. reported that 14‐day continuous ECG patch monitoring detected 20% of AF after Day 8 (Liu et al. 2021).

In Japan, only a limited number of studies have explored the prevalence of silent AF using long‐term patch ECG monitoring. Okubo et al. performed a multicenter randomized prospective clinical study (MYBEAT Trial), including patients aged 65 years or older (mean age 73.2 ± 6.5 years) with one or more of the following risk factors: hypertension, diabetes, heart failure, ischemic heart disease, stroke, or transient ischemic attack (Okubo et al. 2022). As a result, 5‐day patch ECG monitoring using myBeat detected silent AF in 16 of 150 subjects (10.7%). Their detection rate (10.7%) was twice as high as our detection rate for those aged 75 years and older (5.2%). This difference might be because of varying degrees of risk factors for developing AF The MYBEAT Trial enrolled outpatients who regularly visited medical facilities for various diseases associated with AF development. In contrast, our subjects were the general public undergoing health checkups.

Several such reports have been published worldwide. In 2014, Barrett et al. compared simultaneous ambulatory ECG recordings with conventional 24‐h Holter monitoring and 14‐day adhesive patch monitoring (Zio Patch) in 146 patients referred for evaluation of cardiac arrhythmias (Barrett et al. 2014). The adhesive patch monitor detected 96 arrhythmia events (atrioventricular block, pause, polymorphic ventricular tachycardia, supraventricular tachycardia, ventricular tachycardia, or AF) compared with 61 arrhythmia events using the Holter monitor (*p* < 0.001). However, they did not analyze the AF detection rate (Barrett et al. 2014). In 2015, Turakhia et al. reported a single‐center prospective screening study using the same device (Zio Patch) (Turakhia et al. 2015). Inclusion criteria were age ≥ 55 years and ≥ 2 of the following risk factors: coronary disease, heart failure, hypertension, diabetes, and sleep apnea. In subjects (mean 69.0 ± 6.5 years), AF was detected in four subjects (5.3%; AF burden 28 ± 48%). In 2018, Steinhubl et al. randomized 2659 individuals (mean 72.4 ± 7.3 years) to active home‐based monitoring to start immediately (*n* = 1364) or delayed by 4 months (*n* = 1291) (Steinhubl et al. 2018). The silent AF was identified within 4 months in 3.9% (53/1366) of the immediate group vs. 0.9% (12/1293) in the delayed group. The authors concluded that among individuals at a high risk of AF, immediate monitoring with a home‐based wearable ECG sensor patch, compared with delayed monitoring, resulted in a higher rate of AF diagnosis (Steinhubl et al. 2018). In 2021, Gladstone et al. performed a randomized clinical trial of 856 participants aged 75 years or older (mean age 80.0 ± 4.0 years) with hypertension from outpatient primary care practices (Gladstone et al. 2021). The screening group received a 2‐week continuous ECG patch monitor at baseline and 3 months. Silent AF was detected in 5.3% of the screening group. Their detection rate (5.3%) was very close to our detection rate of 5.2% in patients aged 75 years and older.

What kind of management should be performed for silent AF, such as the one detected in our 7‐day patch ECG monitoring, is an important question. In NOAH‐AFNET 6, patients aged 65 years or older who had device‐detected atrial high‐rate episodes (AHREs) lasting for at least 6 min and who had at least one additional risk factor for stroke (mean 77.5 ± 6.7 years) were randomly assigned in a 1:1 ratio to receive either edoxaban or placebo (Kirchhof et al. 2023). Edoxaban did not significantly reduce the incidence of a composite of cardiovascular death, stroke, or systemic embolism compared with a placebo, but it led to a higher incidence of a composite of death or major bleeding (Kirchhof et al. 2023). It should be noted that the enrollees in this study were patients with device‐detected AHREs, but no AF was detected on the ECG. On the other hand, ARTESIA enrolled patients (CHA_2_DS_2_‐VASc: 3 or higher) who had subclinical AF that was detected by an implanted pacemaker, defibrillator, or cardiac monitor, with at least one episode lasting 6 min or longer but no episodes lasting longer than 24 h (mean 76.8 ± 7.6 years) (Healey et al. 2024). Apixaban was associated with a lower risk of stroke and systemic embolism than aspirin. In the present study, among the 16 subjects with silent AF, the minimum AF burden detected by 7‐day patch ECG monitoring was 0.66 min, the maximum was 3708 min, and the mean was 512 min. Since all of these 16 patients had a CHADS_2_ score of 1 or higher, OAC was initiated in accordance with the guideline, except for one patient who did not present for medical evaluation (Ono et al. 2022). However, whether OAC is useful in our patients, in whom silent AF was detected by long‐term ECG patch monitoring, needs to be verified.

As of October 2023, the population of Japan aged 75 years or older is 20,078,000 (Statistics Bureau of Japan 2024). Assuming that the population aged 75 years and older that we tested is representative of the Japanese average, if all individuals aged 75 years and older undergo a 7‐day patch ECG monitoring, 1,040,000 people will be diagnosed with silent AF.

## 5 Conclusions

We conclude that 7‐day patch ECG monitoring during national insurance health checkup in the general population efficiently detected silent AF in individuals aged 75 years and older.

## Author contributions

M.M. contributed to conceptualization, methodology, formal analysis, and writing – original draft. N.K. and H.S. contributed to conceptualization, formal analysis, and writing – review & editing. K.M. and H.K. contributed to data collection and writing – review & editing. T.S. contributed to conceptualization, methodology, and writing – review & editing. N.T. contributed to conceptualization, methodology, writing – review & editing, and supervision. All authors have read and approved the final manuscript.

## Ethics Statement

The Oita University Research Ethics Committee approved this study (No. 2555).

## Consent

All participants provided written informed consent.

## Conflicts of Interest

The authors declare no conflicts of interest.

## Data Availability

No data available.

## References

[anec70092-bib-0001] Akao, M. , Y.‐H. Chun , H. Wada , et al. 2013. “Current Status of Clinical Background of Patients With Atrial Fibrillation in a Community‐Based Survey: The Fushimi AF Registry.” Journal of Cardiology 61, no. 4: 260–266. 10.1016/j.jjcc.2012.12.002. 23403369

[anec70092-bib-0002] Barrett, P. M. , R. Komatireddy , S. Haaser , et al. 2014. “Comparison of 24‐Hour Holter Monitoring With 14‐Day Novel Adhesive Patch Electrocardiographic Monitoring.” American Journal of Medicine 127, no. 1: 95. 10.1016/j.amjmed.2013.10.003. PMC3882198 24384108

[anec70092-bib-0003] Gladstone, D. J. , R. Wachter , K. Schmalstieg‐Bahr , et al. 2021. “Screening for Atrial Fibrillation in the Older Population: A Randomized Clinical Trial.” JAMA Cardiology 6, no. 5: 558–567. 10.1001/jamacardio.2021.0038. 33625468 PMC7905702

[anec70092-bib-0004] Hayano, J. , and E. Yuda . 2022a. “Enhanced Detection of Abnormalities in Heart Rate Variability and Dynamics by 7‐Day Continuous ECG Monitoring.” Annals of Noninvasive Electrocardiology: The Official Journal of the International Society for Holter and Noninvasive Electrocardiology, Inc 27, no. 1: e12897. 10.1111/anec.12897. 34546637 PMC8739595

[anec70092-bib-0005] Hayano, J. , and E. Yuda . 2022b. “Night‐To‐Night Variability of Sleep Apnea Detected by Cyclic Variation of Heart Rate During Long‐Term Continuous ECG Monitoring.” Annals of Noninvasive Electrocardiology: The Official Journal of the International Society for Holter and Noninvasive Electrocardiology, Inc 27, no. 2: e12901. 10.1111/anec.12901. 34661952 PMC8916582

[anec70092-bib-0006] Healey, J. S. , R. D. Lopes , C. B. Granger , et al. 2024. “Apixaban for Stroke Prevention in Subclinical Atrial Fibrillation.” New England Journal of Medicine 390, no. 2: 107–117. 10.1056/NEJMoa2310234. 37952132

[anec70092-bib-0007] Inoue, H. , A. Fujiki , H. Origasa , et al. 2009. “Prevalence of Atrial Fibrillation in the General Population of Japan: An Analysis Based on Periodic Health Examination.” International Journal of Cardiology 137, no. 2: 102–107. 10.1016/j.ijcard.2008.06.029. 18691774

[anec70092-bib-0008] Iwasaki, Y.‐K. , T. Noda , M. Akao , et al. 2025. “JCS/JHRS 2024 Guideline Focused Update on Management of Cardiac Arrhythmias.” Circulation Journal: Official Journal of the Japanese Circulation Society: CJ240073. 10.1253/circj.CJ-24-0073. 39956587

[anec70092-bib-0009] Kirchhof, P. , T. Toennis , A. Goette , et al. 2023. “Anticoagulation With Edoxaban in Patients With Atrial High‐Rate Episodes.” New England Journal of Medicine 389, no. 13: 1167–1179. 10.1056/NEJMoa2303062. 37622677

[anec70092-bib-0010] Kodani, E. , T. Kaneko , H. Fujii , et al. 2019. “Prevalence and Incidence of Atrial Fibrillation in the General Population Based on National Health Insurance Special Health Checkups–TAMA MED Project‐AF.” Circulation Journal: Official Journal of the Japanese Circulation Society 83, no. 3: 524–531. 10.1253/circj.CJ-18-1038. 30643080

[anec70092-bib-0011] Liu, C.‐M. , S.‐L. Chang , Y.‐H. Yeh , et al. 2021. “Enhanced Detection of Cardiac Arrhythmias Utilizing 14‐Day Continuous ECG Patch Monitoring.” International Journal of Cardiology 332: 78–84. 10.1016/j.ijcard.2021.03.015. 33727122

[anec70092-bib-0012] Okubo, Y. , T. Tokuyama , S. Okamura , et al. 2022. “Evaluation of the Feasibility and Efficacy of a Novel Device for Screening Silent Atrial Fibrillation (MYBEAT Trial).” Circulation Journal: Official Journal of the Japanese Circulation Society 86, no. 2: 182–188. 10.1253/circj.CJ-20-1061. 34148927

[anec70092-bib-0013] Ono, K. , Y.‐K. Iwasaki , M. Akao , et al. 2022. “JCS/JHRS 2020 Guideline on Pharmacotherapy of Cardiac Arrhythmias.” Journal of Arrhythmia 38, no. 6: 833–973. 10.1002/joa3.12714. 36524037 PMC9745564

[anec70092-bib-0014] Statistics Bureau of Japan . 2024. “Current Population Estimates as of October 1, 2023.” https://www.stat.go.jp/english/data/jinsui/2023np/index.html.

[anec70092-bib-0015] Steinhubl, S. R. , J. Waalen , A. M. Edwards , et al. 2018. “Effect of a Home‐Based Wearable Continuous ECG Monitoring Patch on Detection of Undiagnosed Atrial Fibrillation: The mSToPS Randomized Clinical Trial.” JAMA 320, no. 2: 146–155. 10.1001/jama.2018.8102. 29998336 PMC6583518

[anec70092-bib-0016] Turakhia, M. P. , A. J. Ullal , D. D. Hoang , et al. 2015. “Feasibility of Extended Ambulatory Electrocardiogram Monitoring to Identify Silent Atrial Fibrillation in High‐Risk Patients: The Screening Study for Undiagnosed Atrial Fibrillation (STUDY‐AF).” Clinical Cardiology 38, no. 5: 285–292. 10.1002/clc.22387. 25873476 PMC4654330

